# Feedback, fairness, and validity: effects of disclosing and reusing multiple-choice questions in medical schools

**DOI:** 10.1080/10872981.2022.2143298

**Published:** 2022-11-09

**Authors:** Stefan Appelhaus, Susanne Werner, Pascal Grosse, Juliane E. Kämmer

**Affiliations:** a Institute of Medical Sociology and Rehabilitation Science, Charité—Universitätsmedizin Berlin, corporate member of Freie Universität Berlin, Humboldt-Universität zu Berlin, and Berlin Institute of Health, Berlin, Germany; b Department of Radiology and Nuclear Medicine, Universitätsmedizin Mannheim, Heidelberg University, Mannheim, Germany; c Assessment Unit, Charité—Universitätsmedizin Berlin, corporate member of Freie Universität Berlin, Humboldt-Universität zu Berlin, and Berlin Institute of Health, Berlin, Germany; d Dean of Students Office and Department of Neurology, Charité—Universitätsmedizin Berlin, corporate member of Freie Universität Berlin, Humboldt-Universität zu Berlin, and Berlin Institute of Health, Berlin, Germany; e Department of Emergency Medicine, University of Bern, Bern, Switzerland

**Keywords:** Assessment, formative assessment, multiple-choice questions, item reuse, disclosure, feedback, progress testing

## Abstract

**Background:**

Disclosure of items used in multiple-choice-question (MCQ) exams may decrease student anxiety and improve transparency, feedback, and test-enhanced learning but potentially compromises the reliability and fairness of exams if items are eventually reused. Evidence regarding whether disclosure and reuse of test items change item psychometrics is scarce and inconclusive.

**Methods:**

We retrospectively analysed difficulty and discrimination coefficients of 10,148 MCQ items used between fall 2017 and fall 2019 in a large European medical school in which items were disclosed from fall 2017 onwards. We categorised items as ‘new’; ‘reused, not disclosed’; or ‘reused, disclosed’. For reused items, we calculated the difference from their first ever use, that is, when they were new. Differences between categories and terms were analysed with one-way analyses of variance and independent-samples *t* tests.

**Results:**

The proportion of reused, disclosed items grew from 0% to 48.4%; mean difficulty coefficients increased from 0.70 to 0.76; that is, items became easier, *P* < .001, η_p_^2^ = 0.011. On average, reused, disclosed items were significantly easier (*M* = 0.83) than reused, not disclosed items (*M* = 0.71) and entirely new items (*M* = 0.66), *P* < .001, η_p_^2^ = 0.087. Mean discrimination coefficients increased from 0.21 to 0.23; that is, item became slightly more discriminating, *P* = .002, η_p_^2^ = 0.002.

**Conclusions:**

Disclosing test items provides the opportunity to enhance feedback and transparency in MCQ exams but potentially at the expense of decreased item reliability. Discrimination was positively affected. Our study may help weigh advantages and disadvantages of using previously disclosed items.

## Introduction

With the continuing trend towards competency-based curricula and formative assessment in medical education, the expectations for assessment have been growing. Rather than just reliably measuring students’ performance, assessment also has to provide feedback about the learners’ strengths and weaknesses, enhance learning, and steer their learning process, often summarised by the term test-enhanced learning [1–3]. To fulfil these expectations, many new assessment formats such as objective structured clinical examinations and short-answer questions have been developed [4]. Unfortunately, the development and realisation of these new assessment formats require many resources. Thus, written examinations using multiple-choice questions (MCQs) are still a key element in the assessment of medical students across the world [5]. To use MCQs in frequent formative assessments, items are needed in great numbers. To achieve this goal many medical schools around the world reuse items, as resources and personnel are usually limited [5–7]. To ensure that the psychometric properties of MCQs are maintained, examiners go to great lengths to keep items confidential [8]. This strict confidentiality is especially important for summative assessments, such as in high-stakes medical licensure examinations (e.g., the USA Medical Licensure Examination), but it does not allow for providing feedback to examinees. Feedback is, however, one of the crucial properties of formative assessment, as it can foster test-enhanced learning and channel long-term retention of course content [9,10]. Thus, the possibility of providing feedback is one argument for item disclosure.

Further arguments for disclosure include the increased overall transparency of the process of administering exams, the reduced effort required of examiners to ensure test items are kept confidential, the opportunity for examinees to better prepare for the test, and the reduction of student anxiety [11–14]. Conversely, the main argument against disclosure is that examinees may more easily obtain previous original test items so that they will eventually test better than earlier cohorts and, in consequence, compromise reliability [7].

To date, evidence for the latter argument has been scarce and contradictory [11]. For example, Yang et al. investigated effects of disclosure of items, answers, and performance data in South Korea’s medical licensing examination [15]. They found no significant changes in student performance, pass rates, or item psychometrics but they did not reuse previously disclosed items. Herskovic disclosed items by discussing them after the exam with examinees without giving them copies of the items to keep [16]. He found no relevant change in psychometric parameters when items were reused in following exams. Wood stated that repeat candidates did not have better scores for those items they had answered before compared to unknown items [17]. However, the sample size in both studies (Herskovic: 197 reused items, number of examinees not stated; Wood: 26 items, 130 examinees) was rather small. Joncas et al. exclusively studied the changes in psychometric parameters over a period of five years during which 1,629 items were reused up to four times in a Canadian medical school without official disclosure of items. They showed that each reuse of items led to a decline in difficulty and discrimination [6]. This deterioration of item psychometrics supports the assumption that it is common for examinees in many medical schools to try to obtain exam questions from previous examinees [7]. It remains unclear whether official disclosure of items would have an additional effect on item psychometrics if students apparently have access to original items anyway.

Hence, given the widespread use of MCQ items and the inconsistent evidence concerning the effects of disclosure and reuse, a more systematic analysis of the problem is obviously a desideratum. Our study addresses this concern, making use of a quasi-experimental setting in one of the largest medical faculties in Europe. For reasons of practicality and transparency, our medical school implemented a radical policy change regarding the disclosure of items. Changing from a previously very restrictive practice that allowed no disclosure of items whatsoever, from fall 2017 students were allowed to take home their tests and were provided with the answer keys, thus making it much easier to provide feedback. Obviously, subsequent students faced no challenge obtaining the original items. Before fall 2017, students had to turn in their tests so that they could, at best, only try to memorise the items, and answer keys were not published. Because of this policy change, the percentage of reused, disclosed items gradually grew from 0% to 48% per exam between fall 2017 and fall 2019.

This policy change offers an exceptional opportunity to examine the effect of disclosure and reuse of test items by comparing the results of exams prior to and after the disclosure of test items. Such a constellation comes close to an experimental setting in which the results prior to disclosure act as the control group. Using this methodological approach, we analysed potential differences in item psychometrics between (a) reused, disclosed items, (b) reused, not disclosed items, and (c) entirely new items. To better assess the effect of disclosure, we analysed the changes in item psychometrics of reused (both, disclosed and not disclosed) items compared to their first ever use (i.e., when they were new items). In line with previous, smaller scale research [6], we hypothesised that items would become easier to solve correctly and less discriminating on reuse and even more so on reuse after disclosure, as students theoretically had no access to original test items before this policy change and would now possibly obtain them more easily.

## Methods

### Study setting

The study was conducted at *Charité – Universitätsmedizin Berlin*, one of Europe’s largest medical schools with approximately 4,500 medical students. At the end of each term (two terms per year), all students from their first to their fifth year take an end-of-term exam that uses exclusively one-best-answer MCQs. As the curriculum is organised in a modular fashion, each exam is interdisciplinary, covering basic sciences and clinical disciplines. For the time our study covers, each exam comprised between 40 and 120 items, depending on the term. Each exam was offered twice per term; items used in the first exam could not be used in the second. All exams were paper based. Prior to 2017, exam copies were collected after the exam and answer keys were not published. Since 2017, students have been allowed to take their individual copies home and answer keys have been published online.

All exams are developed according to a standardised procedure: The learning aims are selected randomly but are nevertheless based on a blueprint so that the selection of topics tested in the exam is representative of the whole module. For up to 80% of these selected learning aims, test items that have already been used are randomly selected from a database containing more than 20,000 items developed by faculty over almost a decade. The items for the remaining 20% of the learning aims are developed entirely anew by faculty members. To ensure high-quality exams, items are written only by experts in their respective fields and new items and final exams are proofread by at least two faculty members and one specialist responsible for linguistic and formal quality control of test items. In a post-examination review, students can report potentially flawed questions and the Board of Examiners may change scoring.

Exams at *Charité* are graded according to the principles of the German national licensing exams. Exam grades range between 1 and 5, with 1 being the best and 4 indicating the lowest passing grade. With a grade of 5, students fail the exam. The passing score is 60%. The exam can be considered high stakes as students are not allowed to further pursue their studies without passing the exam; yet, they have up to six tries to do so.

### Study methods

We included all exams used in the five terms between fall 2017 and fall 2019. Note that we did not include the terms after 2019 because assessment practices changed in the course of the COVID-19 pandemic starting in 2020. The exams in fall 2017 were the first to be disclosed. As these exams did not include reused, disclosed items, we used this examination period as a baseline. Only the four examination periods from spring 2018 to fall 2019 included previously disclosed items, naturally in growing proportions.

We assessed the number of participants as well as item difficulty and discrimination coefficients. Item difficulty was determined as the number of students who answered the question correctly divided by the number of all participating students. Results for difficulty range between 0 and 1 with higher values indicating easier items. According to classic test theory, item difficulty coefficients should ideally range from 0.4 to 0.8 [18]. Discrimination was estimated as a Pearson correlation coefficient of students’ performance on the item with their overall score in the exam [18]. Results for discrimination range between −1 and 1 with higher values representing a higher degree of discrimination. Möltner et al. considered a discrimination coefficient of 0.2 or higher acceptable for MCQ items [18]. For difficulty coefficients of 1, it is not possible to calculate discrimination coefficients.

We grouped items according to the date of their last use in an exam into one of the following three categories:

*New*: Never used before this exam.

*Reused, not disclosed*: Last used before disclosure of exam content (i.e., last used in spring 2017 or earlier).

*Reused, disclosed*: last used after disclosure of exam contents (i.e., last used in fall 2017 or later).

We excluded items if the post-examination review deemed them to be inadequate. Note that items may have been used more than once in the study period; in this case they were included for each use in the respective category.

### Analysis

To assess changes in overall mean item psychometrics, we ran two one-way analyses of variance (ANOVAs) with term (fall 2017, spring 2018, fall 2018, spring 2019, and fall 2019) as independent variable and difficulty and discrimination coefficients as dependent variables. To assess differences in item psychometrics between item groups, we ran two one-way ANOVAs with item group as independent variable and difficulty and discrimination coefficients as dependent variables.

To assess the relative impact of reuse without disclosure versus reuse after disclosure on item difficulty and discrimination, we calculated the differences in difficulty and discrimination coefficients (a) between an item’s first use (i.e., when it was a new item) and when it was reused (delta reused, not disclosed – new), and (b) between an item’s first use and when it was reused and disclosed (delta reused, disclosed – new). We entered these differences into an independent-samples *t* test.

Whenever ANOVA results were significant, Bonferroni post hoc tests were used to assess differences between groups. Effect sizes for ANOVAs were calculated as η_p_^2^. We follow Cohen’s [19] definition of benchmarks for small (η_p_^2^ = 0.01), medium (η_p_^2^ = 0.06), and large (η_p_^2^ = 0.14) effects. Effect sizes for *t* tests were calculated as Cohen’s *d* with defined benchmarks for small (*d* = 0.2), medium (*d* = 0.5), and large (*d* = 0.8) effects. All calculations were done using IBM SPSS 25 [20] and figures were created using GraphPad Prism 9 [21].

## Results

**Figure 1.**
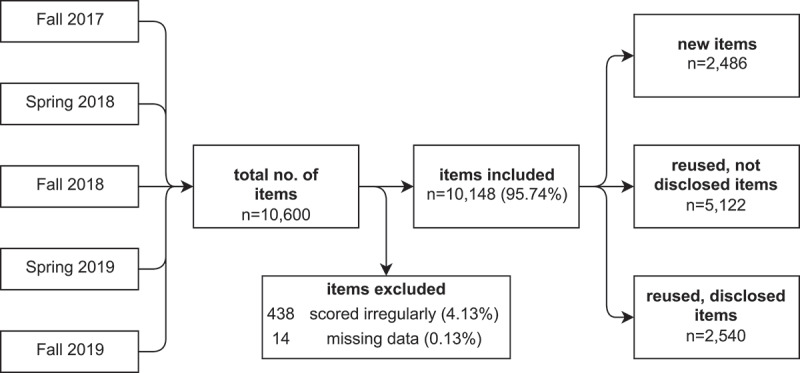
**Overview of origin and types of items**. We analysed all exams conducted between the decision to disclose multiple-choice questions in future examinations in fall 2017 and fall 2019. Fall 2017 was the first exam disclosed to examinees but without reuse of previously disclosed items, thus constituting our baseline for comparison with the exams from spring 2018 to fall 2019, which included reused, previously disclosed items in growing proportions.

We included the results of 199 exams with 23,507 participants and 10,148 items in our analyses (see Figure 1 for an overview of items). Percentage of repeat examinees in the study period was 4.8%.

**Figure 2.**
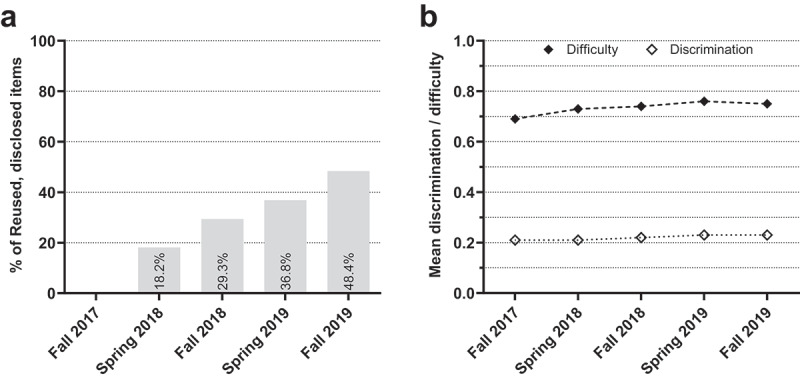
**Item psychometrics over terms**. (a) Proportion of reused, disclosed items across terms. Note that fall 2017 served as baseline without any disclosed items. (b) Mean difficulty and discrimination coefficients, across all item groups, per term.

Although the percentage of reused, disclosed items steadily increased up to 48.4% in fall 2019 (Figure 2a), item psychometrics varied only to a small degree between terms: Mean difficulty coefficients ranged from 0.70 in fall 2017 to 0.76 in spring 2019, *F*(4, 10,143) = 27.03, *P* < .001, η_p_^2^ = 0.011 (Figure 2b). Mean discrimination coefficients ranged from 0.21 in fall 2017 to 0.23 in spring 2019, *F*(4, 9,734) = 4.25, *P* = .002, η_p_^2^ = 0.002 (Figure 2b). Including item groups as an additional independent variable in the analyses revealed that only reused, not disclosed items slightly changed in difficulty over terms. A detailed overview of this additional analysis and all other results can be found in Supplement Table 1.

**Figure 3.**
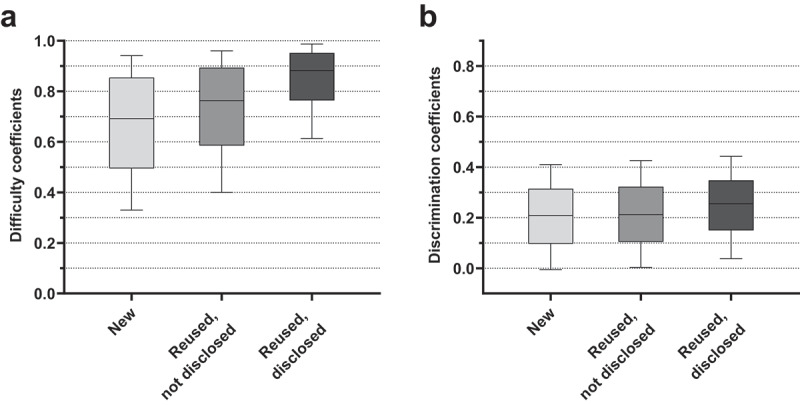
**Boxplot diagram of difficulty and discrimination coefficients between item groups**. Boxplot whiskers mark the 10^th^ and 90^th^ percentiles of the data. (a) Average difficulty coefficients per item group, across terms (including baseline). (b) Average discrimination coefficients per item group, across terms (including baseline).

The ANOVA revealed differences in difficulty of medium effect size between item groups, *F*(2, 10,145) = 483.38, *P* < .001, η_p_^2^ = 0.087. New items were most difficult (*M* = 0.66) followed by reused, not disclosed items (*M* = 0.71) and reused, disclosed items (*M* = 0.83). Bonferroni post hoc tests revealed differences between all groups (all *P* < .001). Mean discrimination coefficients varied between item groups, *F*(2, 9,736) = 49.50, *P* < .001, η_p_^2^ = 0.008, though the effect size was negligible. New items (*M* = 0.20) and reused, not disclosed items (*M* = 0.21) were less discriminating than reused, disclosed items (*M* = 0.25). Bonferroni post hoc tests revealed differences only between reused, disclosed items and the other two item groups (both *P* < .001). Results are presented in Figure 3.

To better assess the effect of disclosure on items, we focused on the groups of reused items and analysed their item psychometrics as compared to their first ever use, that is, when they were new items. Difficulty coefficients decreased by *M* = −0.01 in reused, not disclosed items and increased by *M* = 0.11 in reused, disclosed items, indicating a medium to large effect of disclosure on item difficulty, *t*(4699.79) = −29.69, *P* < .001, *d* = 0.74. Discrimination coefficients increased by *M* = 0.03 in reused, not disclosed items and by *M* = 0.07 in reused, disclosed items, indicating a negligible to small effect of disclosure on item discrimination, *t*(4274.07) = −7.22, *P* < .001, *d* = 0.19.

We did not include students grades and pass rates in our analysis because our medical school applies an automatic adjustment clause used in the German state examinations [22,23]. The latter procedure is meant to level grades across terms and to prevent unusually high failure rates in the high-stakes German medical licensure examination. Mean grades and pass rates are listed in Supplement Table 1. As expected, they are barely affected.

## Discussion

Implementing test-enhanced learning strategies in a curriculum often requires many resources, which may deter medical schools from doing so [1]. By simply disclosing existing items and exams to students, examiners can provide feedback with relatively low effort. Furthermore, withholding exam items from students may lead them to doubt exam transparency and fairness. Because of the need for efficient and frequent assessments under conditions of limited resources, many medical schools rely on item reuse [6,7]. However, the combination of item disclosure and reuse facilitates cheating in the form of content sharing with subsequent examinees, thus potentially jeopardising item validity and exam reliability [7]. Before considering providing feedback through item disclosure, examiners need to know the effects of this measure on item psychometrics. Yet, empirical evidence on the consequences of disclosing exam items has been scarce and mostly contradictory, which reflects the obvious methodological difficulties encountered in addressing such a research question [6,11,15–17]. Our almost unique and quasi-experimental setting, which came about more or less by chance, nevertheless allowed us to investigate the effects of reusing non-disclosed and disclosed test items on item difficulty and discrimination in a large European medical school in a high-income country with a large number of participants and including more than 10,000 test items.

As we hypothesised, our analyses revealed that reused, disclosed items were easier to answer correctly than reused, not disclosed and new items. Also, reused, disclosed items, but not reused, not disclosed items, were answered more accurately (+0.11 in the difficulty coefficient) than when they were used for the first time. These results suggest, first, that the observed change in difficulty coefficients is indeed mostly due to disclosure of items and students using the disclosed items to prepare for their exams. Second, previous measures to keep items confidential seem to have been mostly effective.

Our analyses further revealed, counterintuitively, that reused, disclosed items were slightly more discriminating than reused, not disclosed and new items and also compared to when they were used for the first time. Our finding is in contrast to the results of a recent study [6] that showed a decrease in discrimination with each reuse of an item. The discrepancy might be due to different methods of calculating discrimination coefficients. Whereas Joncas et al. used the item-discrimination index method, we used the point-biserial correlation coefficient, which is generally less affected by decreasing item difficulty [18,24]. Another explanation would be that higher performing students can better memorise a higher number of items, leading to increased discrimination. We conclude that items still discriminate correctly between higher and lower performing students, and, thus, item disclosure seems not to interfere with correct student ranking.

We suspect that the size of the effects item disclosure and reuse have on difficulty and discrimination depends on the size of the item pool from which exam items are drawn. Our medical school’s item bank contains more than 20,000 MCQs altogether, which translates to roughly 2,000 potential items per exam, of which only a few are randomly selected for the end-of-term exam. It seems obvious that it is almost impossible for students to perfectly memorise this many items. Naturally, the smaller an item bank is, the easier it is for students to memorise items and the faster the proportion of disclosed items increases. Thus, the effects of item disclosure may be more pronounced in institutions that do not generate as many test items as our institution has done. To oppose this effect, institutions could attempt to enlarge their item banks. To do so, medical schools have tried innovative approaches such as developing items using artificial intelligence [25] or having students write items as part of the coursework. Especially the latter has been shown to have positive effects on learning, too [26,27].

In sum, our results show that increased transparency and feedback in MCQ exams come at the expense of item reliability. Whether the size of this effect is practically relevant most likely depends on the goals of exams and their underlying philosophy [2]. In high-stakes, career-deciding exams such as medical licensure examinations, even small changes in reliability induced by disclosure and reuse of items may be too big to tolerate. In formative assessment settings in which discrimination and feedback are more important than weeding out students through reliable passing scores, a drop in item psychometrics may be outweighed by the benefits of disclosure and reuse.

Disclosing items for the purpose of providing feedback can take many forms, from simply publishing exams and answer keys to incorporating individualised feedback and explanations on systematic feedback platforms (e.g., [28],). Yet, developing such feedback platforms requires many resources, which may be especially problematic for smaller medical schools. Thus, progress testing may be a viable alternative to frequent assessment and disclosing items [29,30], as progress testing shares many of the advantages of item disclosure, such as better feedback options, increased transparency, and reduced student anxiety, but also the ensuing need for large item banks. Progress testing has the additional advantage of providing systematic individual and longitudinal feedback, which likely entails much richer information than just providing items and answer keys without explanation [31,32].

It is an empirical question whether our results can be generalised to other medical schools with perhaps smaller item banks, different examination schedules, alternative blueprints for their exams, or different methods to prevent leakage of items. Nevertheless, our study could potentially provide a more general lesson regarding the development, use, and reuse of MCQs. It would be rewarding if our study could inspire further studies in various academic settings and in countries with other academic traditions. Finally, it would be of particular interest to investigate whether the effects delineated in our study can be classified as short-term or sustainable effects. To answer the latter question, follow-up studies at our institution covering a longer time span are mandatory. As the assumed positive effects of disclosure, for example, reduced anxiety and test-enhanced learning, have been shown only in other settings, student surveys or correlation with dropout rates and licensure examination results would be interesting for further research.

## Conclusion

Our study provides evidence supporting the argument that item disclosure in combination with item reuse decreases item difficulty and increases discrimination. Thus, disclosure may compromise exam reliability to a moderate extent. Our results may help educators weigh the observed disadvantages of item disclosure against the obvious benefits such as increased transparency, reduced student anxiety, and the opportunity to provide better feedback.

## Supplementary Material

Supplemental Material
